# The Dark Side of Vascular Aging: Noncoding Ribonucleic Acids in Heart Failure with Preserved Ejection Fraction

**DOI:** 10.3390/cells14161269

**Published:** 2025-08-16

**Authors:** Jianning Chen, Xiao Xiao, Charles Zhou, Yajing Zhang, James Rhee, Haobo Li

**Affiliations:** 1 Department of Anesthesia, Critical Care, and Pain Medicine, Massachusetts General Hospital, Boston, MA 02114, USA; chenjianning608@gmail.com (J.C.); xxiao6@mgh.harvard.edu (X.X.); czhou22@mgh.harvard.edu (C.Z.); yzhang188@mgh.harvard.edu (Y.Z.); 2 Harvard Medical School, Boston, MA 02114, USA

**Keywords:** heart failure with preserved ejection fraction, vascular aging, noncoding RNA, endothelial senescence, arterial stiffness, microvascular dysfunction

## Abstract

Heart failure with preserved ejection fraction (HFpEF) represents a growing global public health challenge, now accounting for approximately half of all heart failure cases and often linked to a systemic pathophysiological process in older adults with multiple comorbidities. Despite increasing recognition of the vascular contributions to HFpEF, the precise molecular mechanisms, particularly the role of noncoding Ribonucleic Acids (ncRNAs) in mediating vascular aging and subsequent cardiac dysfunction, remain incompletely understood. This review provides a comprehensive overview of the mechanistic link between vascular aging and HFpEF, with a specific focus on the pivotal roles of ncRNAs in this complex interplay. We delineate the classification of vascular aging, its cellular hallmarks, including endothelial senescence, vascular smooth muscle cell phenotypic switching, and extracellular matrix remodeling, and its systemic implications, such as inflammaging, oxidative stress, and reduced nitric oxide bioavailability. We then detail how these vascular alterations, including increased ventricular afterload and impaired myocardial perfusion due to coronary microvascular dysfunction, contribute to HFpEF pathophysiology. The review extensively discusses recent findings on how diverse classes of ncRNAs, notably microRNAs, long noncoding RNAs, and circular RNAs, along with emerging evidence for PIWI-interacting RNAs, small nuclear RNAs, small nucleolar RNAs, and tRNA-derived small RNAs, regulate these vascular aging processes and serve as molecular bridges connecting vascular dysfunction to heart failure. In conclusion, understanding the regulatory landscape of ncRNAs in vascular aging may reveal novel biomarkers and therapeutic avenues, offering new strategies for precision medicine in HFpEF.

## 1. Introduction

Heart failure (HF) with preserved ejection fraction (HFpEF) is emerging as one of the most prevalent and therapeutically challenging forms of HF [1,2], particularly among aging populations with a high burden of metabolic and inflammatory comorbidities. In contrast to heart failure with reduced ejection fraction (HFrEF), which is typically driven by primary myocardial injury, HFpEF is increasingly understood as a systemic syndrome rooted in vascular dysfunction, chronic low-grade inflammation, and impaired cardiometabolic homeostasis [3]. Importantly, recent research has highlighted that noncoding RNAs (ncRNAs) play critical regulatory roles in both HFrEF and HFpEF pathophysiology, influencing processes ranging from myocardial remodeling and fibrosis in HFrEF to endothelial dysfunction, inflammation, and vascular aging in HFpEF.

At the core of this paradigm lies vascular aging, a dynamic process encompassing endothelial senescence, arterial stiffening, microvascular rarefaction, extracellular matrix (ECM) remodeling, and inflammaging, which collectively contribute to increased cardiac afterload, impaired myocardial perfusion, and diastolic dysfunction [4]. Far from a passive consequence of chronological aging, vascular aging represents an active, multifactorial remodeling process orchestrated by genetic, epigenetic, metabolic, and environmental factors [5]. These alterations disrupt vascular compliance, tissue perfusion, and signaling across the vascular–cardiac axis [6], serving as a foundational pathophysiological substrate for HFpEF [7]. Crucially, many of these changes occur before overt cardiac symptoms, offering opportunities for early intervention and mechanistic insights.

NcRNAs have recently emerged as essential regulators of cardiovascular homeostasis and aging. Among the various ncRNAs, microRNAs (miRNAs) modulate gene expression post-transcriptionally, playing established roles in endothelial function, inflammation, and myocardial hypertrophy. Long noncoding RNAs (lncRNAs) and circular RNAs (circRNAs) regulate transcriptional and post-transcriptional processes, influencing vascular smooth muscle plasticity, fibrosis, and cellular senescence. Moreover, emerging classes of ncRNAs, including PIWI-interacting RNAs (piRNAs), tRNA-derived small RNAs (tsRNAs), small nuclear RNAs (snRNAs), small nucleolar RNAs (snoRNAs), enhancer RNAs (eRNAs), Y RNAs, small Cajal body-specific RNAs (scaRNAs), and vault RNAs, have demonstrated significant regulatory potential in vascular biology and cardiovascular aging. Understanding their distinct roles may uncover novel diagnostic markers and therapeutic targets [8].

In this review, we provide a comprehensive overview of how vascular aging contributes to the pathogenesis of HFpEF and emphasize the multifaceted roles of diverse ncRNA classes in regulating these processes. We first describe the anatomical and cellular hallmarks of vascular aging and its systemic implications. We then examine how distinct ncRNAs mediate endothelial dysfunction, smooth muscle plasticity, and ECM remodeling, linking vascular injury to diastolic HF. Finally, we discuss the translational potential of ncRNAs as diagnostic biomarkers and therapeutic targets, offering a framework for future precision medicine in age-related cardiovascular disease.

## 2. The Landscape of Vascular Aging in Cardiovascular Disease

Vascular aging is a fundamental biological process and a critical determinant of cardiovascular risk, contributing to a broad spectrum of diseases, including hypertension, atherosclerosis, coronary microvascular dysfunction, and HFpEF. It involves active molecular and cellular remodeling events, governed by genetic, epigenetic, metabolic, and environmental factors. These changes progressively impair vascular structure, elasticity, and homeostatic function.

### 2.1. Classification of Vascular Aging: Macrovascular vs. Microvascular and Physiological vs. Pathological

Vascular aging can be analyzed along two axes: that of anatomical scale (macrovascular versus microvascular) and biological trajectory (physiological versus pathological), each with distinct structural, functional, and clinical implications. The macrovascular system typically refers to large arteries and veins with diameters greater than 100 μm, while microvascular system includes arterioles, capillaries, and venules generally less than 100 μm in diameter. This anatomical distinction underpins divergent mechanisms and clinical manifestations of aging in different vascular beds.

Macrovascular aging predominantly involves large, elastic conduit arteries (e.g., the aorta, carotid, and femoral vessels). With advancing age, fragmentation of elastin fibers, increased collagen cross-linking, and medial calcification combine to reduce arterial compliance. Clinically, these changes manifest as elevated pulse wave velocity (PWV) and loss of Windkessel buffering, leading to increased systolic blood pressure and widened pulse pressure. The premature return of reflected pressure waves augments left ventricular afterload, driving concentric hypertrophy and impairing diastolic filling, particularly under stress or in the presence of hypertension, chronic kidney disease, or diabetes. Imaging modalities, such as carotid–femoral PWV, ultrasound-based distensibility, and magnetic resonance elastography, provide robust, noninvasive measures of macrovascular stiffness and have prognostic value for cardiovascular events in older adults [9]. In contrast, microvascular aging implicates arterioles, capillaries, and the precapillary sphincter networks. Pathological hallmarks include endothelial rarefaction, basement membrane thickening, pericyte loss, and impaired nitric oxide (NO)–mediated vasodilation. These changes disrupt tissue autoregulation, heighten vasoconstrictive tone via endothelin-1 upregulation, and compromise perfusion reserve. Metabolically demanding organs, such as the myocardium, brain, and renal cortex, are particularly susceptible to microcirculatory insufficiency. In HFpEF patients and aged rodent models, histological analyses reveal reduced capillary density and increased intercapillary distance in the myocardium, correlating with diastolic dysfunction and exercise intolerance [10]. Emerging imaging techniques, such as contrast-enhanced ultrasound, cardiac magnetic resonance perfusion, and positron emission tomography, allow quantification of myocardial blood flow and have elucidated the prognostic significance of microvascular rarefaction in age-related HF [11].

Biological vascular aging may follow a physiological trajectory, characterized by gradual, homeostatic remodeling in the absence of overt disease, that preserves sufficient compliance and perfusion for routine metabolic demands. In healthy older individuals, compensatory upregulation of endothelial NO synthase (eNOS) and increased antioxidant defense (e.g., superoxide dismutase activity) mitigate oxidative stress, maintaining endothelial integrity and preventing maladaptive remodeling. By contrast, pathological vascular aging is accelerated by classical cardiovascular risk factors, including hyperglycemia, inducing advanced glycation end-product (AGE) cross-linking of extracellular matrix, dyslipidemia promoting endothelial foam cell formation and low-grade inflammation, and smoking exacerbating oxidative damage and reducing NO bioavailability. These stressors drive unchecked collagen deposition, matrix metalloproteinase (MMPs) activation, and vascular smooth muscle cell (VSMC) phenotypic switching, culminating in severely impaired vasomotor control, heightened arterial stiffness, and microvascular rarefaction. Pathological aging is a strong predictor of cardiovascular morbidity and mortality and a principal etiology of HFpEF.

Distinguishing physiological from pathological vascular aging has direct translational relevance. In asymptomatic older individuals, identification of subclinical pathological changes, via elevated PWV, reduced flow-mediated dilation, or diminished microvascular reserve, can enable targeted lifestyle or pharmacologic interventions aimed at halting progression toward HFpEF. Conversely, in established HFpEF, multimodal assessment of both macrovascular stiffness and microvascular function offers deeper phenotyping, informing personalized management strategies that combine vascular-directed therapies (e.g., nitrates, endothelial protective agents) with diastolic-function modulators.

### 2.2. Cellular Hallmarks: Endothelial Senescence, VSMC Phenotype Switching, and ECM Remodeling

The vascular wall is a highly organized multicellular structure composed of distinct layers and specialized cell types. In large conduit arteries (macrovasculature), the tunica intima is lined with endothelial cells (ECs) that regulate vascular tone, permeability, and inflammation. The tunica media is dominated by VSMCs, which maintain contractility and vessel integrity, and the tunica adventitia contains fibroblasts, immune cells, and ECM components that provide structural support. In contrast, the microvasculature, comprising arterioles, capillaries, and venules, is composed primarily of ECs and pericytes, with a sparse or absent medial layer. These anatomical and cellular distinctions underpin the heterogeneous aging trajectories and distinct pathophysiological consequences observed in macrovascular and microvascular compartments. Vascular aging manifests at the cellular level through maladaptive changes in ECs, VSMCs, and the ECM, which together compromise vessel integrity and function.

Endothelial Senescence. A pivotal early event in vascular aging is the onset of EC senescence, marked by irreversible cell cycle arrest, diminished NO bioavailability, and adoption of a proinflammatory, prothrombotic secretory profile. Senescent ECs downregulate eNOS and upregulate adhesion molecules such as VCAM-1 and ICAM-1, fostering leukocyte adhesion and local inflammation. The resulting senescence-associated secretory phenotype (SASP) releases cytokines (e.g., IL-6, IL-8), chemokines, and MMPs that propagate dysfunction in neighboring cells [12]. In murine models, EC-specific deletion of SIRT1 or eNOS accelerates vascular stiffening and precipitates HFpEF-like diastolic dysfunction, underscoring the causal role of EC aging in HF [13]. Clinically, elevated circulating EC-derived microparticles, a biomarker of endothelial injury, increase with chronological age and predict higher PWV and cardiovascular mortality in population studies [14].

VSMC Phenotypic Switching. In response to hemodynamic stress, oxidative insults, and inflammatory mediators, VSMCs transition from a contractile phenotype, characterized by high expression of smooth muscle actin and contractile myosin, to a synthetic phenotype with increased proliferative and migratory capacity. Synthetic VSMCs secrete excessive ECM components (collagens I/III, fibronectin), proinflammatory cytokines, and MMPs, driving intimal thickening, elastin fragmentation, and vascular calcification. The net effect is loss of medial elasticity and predisposition to increased arterial stiffness.

ECM Remodeling. The culmination of cellular aging processes is aberrant ECM turnover. Aging vessels exhibit elevated activity of lysyl oxidase (LOX) and tissue transglutaminases that enzymatically cross-link collagen and elastin fibers, rendering the matrix stiff and noncompliant. Concurrently, elastase and MMP-mediated elastin degradation produce fragmented elastic lamellae, further impairing vascular recoil. These compositional and structural changes increase pulse pressure and augment left ventricular afterload, while similar fibrotic remodeling in the myocardium contributes to diastolic dysfunction. Importantly, matrix cross-linking by AGEs in hyperglycemic states further accelerates stiffness, bridging the molecular pathology of metabolic disease with HFpEF progression.

Together, endothelial senescence, VSMC phenotype switching, and ECM remodeling constitute interdependent cellular hallmarks of vascular aging. Their combined impact on vessel compliance, microvascular perfusion, and inflammation results in increased cardiac workload and diastolic impairment, linking age-related vascular deterioration directly to HFpEF pathogenesis. Investigation into the molecular drivers of these processes offers promise for targeted therapies aimed at rejuvenating vascular function and mitigating HF risk.

### 2.3. Systemic Implications: Inflammaging, Oxidative Stress, Arterial Stiffness, and Reduced NO Bioavailability

Vascular aging at the cellular level propagates a cascade of systemic alterations that collectively undermine cardiovascular resilience and precipitate end-organ injury. Among these, inflammaging, oxidative stress, arterial stiffening, reduced NO bioavailability, and lymphatic dysfunction stand out as interconnected drivers of pathology.

Inflammaging. With advancing age, the vasculature is exposed to persistently elevated levels of proinflammatory mediators, including interleukin-6 (IL-6), tumor necrosis factor-α (TNF-α), and C-reactive protein (CRP), that together define “inflammaging.” Beyond mere biomarkers, these cytokines actively perpetuate EC activation, promoting increased expression of adhesion molecules (VCAM-1, ICAM-1) and chemokines that facilitate leukocyte extravasation and vascular wall inflammation. Chronic IL-1β signaling worsens this milieu by upregulating MMPs and degrading the basement membrane, thereby accelerating adverse arterial remodeling. In aged rodent models, pharmacologic blockade of IL-6 or IL-1β receptors restores endothelium-dependent vasodilation and lowers PWV, underscoring the therapeutic potential of anti-inflammatory strategies in combating age-related vascular dysfunction [15,16].

Oxidative Stress. Mitochondrial dysfunction and upregulation of NADPH oxidase isoforms (NOX1/2/4) converge to generate excessive reactive oxygen species (ROS), which in turn oxidize lipids, proteins, and nucleic acids within the arterial wall. ROS-mediated inactivation of eNOS and direct scavenging of NO reduce endothelium-dependent vasodilatory capacity, fostering vasoconstriction and VSMC proliferation. Concurrent downregulation of antioxidant defenses, such as superoxide dismutase and glutathione peroxidase, exacerbates redox imbalance and further potentiates inflammatory signaling via NF-κB activation. Interventions that restore mitochondrial biogenesis (e.g., PGC-1α agonists) or inhibit NOX activity have demonstrated efficacy in reducing vascular ROS production and improving arterial compliance in preclinical studies, highlighting oxidative stress as a modifiable component of vascular aging [17].

Arterial Stiffness. The structural sequelae of ECM remodeling and VSMC phenotypic switching manifest as increased arterial stiffness, quantified clinically by PWV and central pulse pressure measurements. Elastin fragmentation, driven by elastase and MMP overactivity, and collagen cross-linking (mediated by lysyl oxidase and AGEs in hyperglycemic states) elevate systolic load on the left ventricle. Longitudinal human cohort studies have consistently shown that each 1 m/s increment in carotid-femoral PWV corresponds to a 15% increase in HFpEF incidence and a 20% increase in cardiovascular mortality [18]. These findings highlight arterial stiffness as an active contributor, rather than just a passive marker, in the development of HFpEF.

NO Bioavailability. A unifying mechanistic link between inflammaging, oxidative stress, and arterial stiffness is the progressive reduction in NO bioavailability. Age-related downregulation of eNOS expression and phosphorylation reduces NO synthesis, while increased ROS accelerates NO degradation, shifting the vascular milieu toward vasoconstriction, platelet aggregation, and inflammation. Dysfunction of the NO–cGMP–PKG axis impairs myocardial relaxation by promoting cardiomyocyte hypertrophy and interstitial fibrosis. Pharmacologic agents that boost NO signaling, such as soluble guanylate cyclase stimulators and phosphodiesterase-5 inhibitors, have shown promise in small HFpEF trials by improving exercise tolerance and diastolic function [19].

Lymphatic Dysfunction. Emerging evidence highlights a crucial role for cardiac lymphatic dysfunction in the pathogenesis of HFpEF and aging hearts. The cardiac lymphatic system is essential for maintaining myocardial homeostasis through efficient clearance of interstitial fluids and inflammatory mediators. Recent studies reveal significant structural and functional impairments of cardiac lymphatics in aged and HFpEF animal models, characterized by decreased lymphatic endothelial cell (LEC) density, disrupted vessel integrity, and compromised lymphatic drainage capacity [20]. Guo et al. demonstrated that defective branched-chain amino acid (BCAA) catabolism within cardiac LECs contributes substantially to lymphangiogenic defects and subsequent lymphatic dysfunction [21]. Mechanistically, impaired BCAA catabolism promotes ligand-independent phosphorylation of VEGFR3 via Src kinase, disrupting receptor trafficking to the cell membrane and reducing downstream Akt signaling, glucose metabolism, and lymphangiogenesis. Remarkably, therapeutic stimulation of lymphangiogenesis through selective VEGFR3 activation (using engineered VEGFC^C156S) significantly restores cardiac lymphatic integrity and ameliorates key pathological features of HFpEF, including myocardial fibrosis, diastolic dysfunction, and exercise intolerance. These findings underscore lymphatic endothelial metabolic homeostasis as a novel and promising therapeutic target in mitigating HFpEF progression associated with vascular aging [21].

Integration and Clinical Relevance. Together, inflammaging, oxidative stress, arterial stiffening, NO depletion, and lymphatic dysfunction create a feed-forward loop that transforms vessels into maladaptive contributors to cardiac dysfunction. Recognizing these systemic alterations as interdependent facets of vascular aging elevates them from correlates to causative targets in HFpEF pathogenesis. Critically, many of these processes are modulated by ncRNAs positioned at the nexus of vascular–cardiac aging. Subsequent sections will explore how specific miRNAs, lncRNAs, and circRNAs fine-tune these systemic pathways and offer novel biomarkers and therapeutic entry points for age-related cardiovascular disease.

## 3. Vascular Aging in the Pathogenesis of HFpEF

Vascular aging has emerged as a pivotal contributor to the pathophysiology of HFpEF, particularly among older individuals with comorbidities such as hypertension, diabetes, and obesity. HFpEF is now increasingly understood as a systemic syndrome originating from chronic vascular dysfunction and persistent low-grade inflammation.

### 3.1. Arterial Stiffness and Increased Ventricular Afterload

Progressive arterial stiffening is a defining manifestation of vascular aging and a critical determinant of afterload in HFpEF. With advancing age, and exacerbated by comorbid conditions such as hypertension, diabetes, and chronic kidney disease, large elastic arteries undergo profound structural remodeling. Fragmentation and thinning of elastin lamellae, driven by enhanced elastase and MMPs activity, diminish the elastic recoil essential for Windkessel function. Concurrently, increased synthesis and cross-linking of collagen types I and III, mediated by upregulation of lysyl oxidase and formation of AGEs, further reduce arterial compliance. VSMCs also contribute by proliferating and synthesizing ECM under the influence of angiotensin II, TGF-β, and mechanical stretch, leading to medial hypertrophy and luminal narrowing. Functionally and clinically, these changes are quantified by carotid–femoral PWV, the gold-standard measure of aortic stiffness, and by central aortic pressure assessments. Elevated PWV correlates with and independently predicts the incidence of HFpEF and cardiovascular mortality across diverse populations, including the Framingham and Gutenberg cohorts. Mechanistic studies have demonstrated that the early return of reflected pressure waves, occurring when pulse wave velocity surpasses the duration of systole, augments central systolic pressure by up to 20 mmHg, thereby increasing left ventricular wall stress and myocardial oxygen consumption [22].

Chronically elevated afterload induces concentric left ventricular remodeling, characterized by increased wall thickness and reduced chamber compliance. This hypertrophic response initially preserves systolic function but progressively impairs passive diastolic filling, raising end-diastolic pressures for any given volume. Animal models of targeted elastin degradation or cross-linking induction recapitulate this phenotype, exhibiting increased left ventricular mass, impaired relaxation on pressure–volume loop analysis, and exercise intolerance, features analogous to human HFpEF.

From a therapeutic standpoint, interventions that mitigate arterial stiffening have shown promise in delaying or reversing diastolic dysfunction. Pharmacologic agents, including angiotensin receptor blockers, aldosterone antagonists, and novel AGE breakers, reduce collagen cross-linking and VSMC activation, leading to measurable improvements in PWV and central pulse pressure. Nonpharmacologic strategies, such as structured aerobic exercise and dietary nitrate supplementation, enhance endothelial function, increase elastin integrity, and partially restore compliance. Collectively, these studies highlight arterial stiffness as more than just an indicator of vascular aging; it serves as a treatable contributor to elevated ventricular afterload and the development of HFpEF.

### 3.2. Coronary Microvascular Dysfunction and Impaired Myocardial Perfusion

Coronary microvascular dysfunction is now recognized as a pivotal downstream manifestation of vascular aging, present in up to 75% of patients with HFpEF. Aging, compounded by persistent low-grade inflammation (inflammaging), triggers EC senescence, oxidative damage, and inflammation around blood vessels, all of which together compromise the ability of small arteries and capillaries to dilate in response to endothelial signals. Reduced NO bioavailability, driven by eNOS downregulation and ROS scavenging, alongside upregulation of vasoconstrictors such as endothelin-1 and angiotensin II, diminishes coronary flow reserve. This hemodynamic deficit is quantifiable via positron emission tomography and cardiac magnetic resonance perfusion imaging. HFpEF cohorts typically exhibit coronary flow reserve values below 2.0, directly correlating with exercise intolerance, elevated left ventricular filling pressures, and worse New York Heart Association functional class. Structurally, aging precipitates capillary rarefaction and basement membrane thickening in the myocardium, as evidenced by histological analyses of HFpEF explants and aged animal models. A 20–30% reduction in capillary density not only compromises oxygen delivery under stress but also fosters a shift from fatty acid oxidation toward glycolytic metabolism, exacerbating energetic inefficiency [23]. Subclinical ischemia, even in the absence of epicardial obstructive disease, triggers patchy interstitial fibrosis via activation of cardiac fibroblasts and upregulation of profibrotic mediators such as TGF-β1. The resultant increase in myocardial stiffness directly contributes to diastolic dysfunction, linking microvascular aging to HFpEF pathogenesis. Therapeutic strategies that restore microvascular health, such as SGLT2 inhibitors, which reduce oxidative stress and improve endothelial function, and soluble guanylate cyclase stimulators, which enhance NO–cGMP signaling, have demonstrated improvements in coronary flow reserve and diastolic performance in early-phase clinical trials.

### 3.3. The Vascular–Cardiac Axis: Inflammatory, Hemodynamic, and Metabolic Crosstalk

The progression from vascular aging to HFpEF is orchestrated by a multifaceted vascular–cardiac axis in which inflammatory, hemodynamic, and metabolic signals converge to remodel both vessel and myocardium. At the heart of this axis lies the SASP of aged ECs and VSMCs, characterized by the sustained release of proinflammatory cytokines (e.g., TNF-α, IL-6), chemokines, and growth factors. These mediators engage canonical pathways, such as NF-κB, JAK/STAT, and TGF-β/Smad, to amplify endothelial barrier breakdown, leukocyte adhesion, and activation of cardiac fibroblasts, thereby laying the groundwork for myocardial fibrosis and stiffening. Simultaneously, age-related decline in NO bioavailability, caused by reduced eNOS, oxidative scavenging, and decreased cofactor levels, impairs the NO–cGMP–PKG signaling cascade in both vascular and myocardial cells. In vessels, this shift favors vasoconstriction and augments ROS production, further fueling SASP signaling. In cardiomyocytes, blunted PKG activity diminishes titin phosphorylation, increasing passive stiffness and impairing relaxation. Hemodynamically, the combination of arterial stiffening and microvascular dysfunction elevates pulsatile and mean loading on the left ventricle, while metabolic derangements, such as a shift toward glycolysis in ischemic myocardium, reduce energetic efficiency and exacerbate diastolic impairment. These interdependent processes culminate in disrupted ventricular–vascular coupling: the increased pulsatile load imposed by stiff arteries is met by a heart that cannot augment relaxation or perfusion, leading to elevated filling pressures, reduced stroke volume reserve, and exercise intolerance. Clinically, this manifests as exertional dyspnea, pulmonary congestion, and preserved ejection fraction, hallmarks of HFpEF. Importantly, interventions that target components of this axis, ranging from anti-inflammatories (e.g., IL-6 antagonists) to NO-enhancing agents (e.g., soluble guanylate cyclase stimulators) and metabolic modulators (e.g., SGLT2 inhibitors), have demonstrated improvements in diastolic function and symptom burden in early HFpEF studies, underscoring the axis’s therapeutic relevance [24]. Collectively, these insights advocate for integrated strategies that simultaneously address inflammation, hemodynamics, and metabolism to interrupt the vascular–cardiac crosstalk at its roots.

## 4. Noncoding RNAs as Regulators of Vascular Aging

ncRNAs have emerged as central regulators of vascular homeostasis and disease, integrating diverse stress signals to modulate endothelial function, VSMC phenotype, ECM remodeling, and chronic inflammation. Here, we survey seven major ncRNA classes, miRNAs, lncRNAs, circRNAs, piRNAs, snRNAs, snoRNAs, and tsRNAs, and highlight the evidence connecting each factor to the molecular processes underlying vascular aging and HF development. Depending on the context, ncRNAs exert dualistic roles in driving both injury and repair as critical regulators of endothelial function and fibrosis. As we will review, their dysregulation can precipitate microvascular rarefaction and elevated peripheral resistance, augmented left ventricular afterload, and diastolic dysfunction characteristic of HFpEF. Similarly, ncRNAs underlie the maladaptive VSMC plasticity that is seen in HF, which increases PWV, left ventricular wall stress, concentric hypertrophy, and diastolic dysfunction. As such, ncRNAs not only serve as mechanistic biomarkers of vascular aging but also represent promising therapeutic targets for interrupting the vascular–cardiac axis that underlies age-related HF.

### 4.1. MiRNA

**Figure 1:**
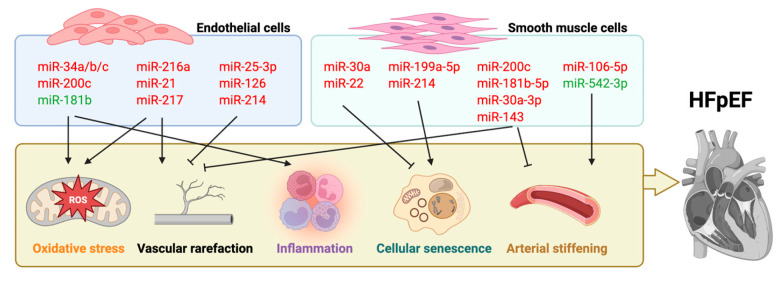
The role of miRNAs in endothelial cells and smooth muscle cells in vascular aging and HFpEF. Created in Biorender. Chen, J. (2025) https://BioRender.com/h8wxsj2.

MicroRNAs (miRNAs) orchestrate virtually every facet of vascular aging by modulating EC phenotypes through finely tuned post-transcriptional control of stress, survival, and differentiation pathways (Table 1 and Figure 1). In aged ECs, up-regulation of miR-34a/b/c and miR-200c suppresses key antioxidant defenses (SIRT1, SIRT3, ZEB1), fueling ROS accumulation and triggering SASP [25]. Concurrently, down-regulation of protective miR-181b exacerbates MAPK-mediated oxidative injury, while pro-senescent miR-216a, miR-21, and miR-217 amplify inflammatory signaling via NF-κB activation and impair NO bioavailability through eNOS repression, thereby driving endothelial dysfunction and microvascular rarefaction [24,26,27]. Importantly, miR-25-3p and miR-126 can exert reparative effects, enhancing VEGFR-2 and HIF-1α signaling to promote angiogenesis, and miR-214 delivered in exosomes from young ECs bolsters endothelial repair, underscoring the dualistic, context-dependent roles of miRNAs in vascular homeostasis [28,29]. In VSMCs, miRNAs regulate autophagy, oxidative stress, and phenotypic switching to govern vessel remodeling (Table 2 and Figure 1). Rapamycin-responsive miR-30a and palmitate-induced miR-22 delay senescence and suppress the synthetic phenotype via Beclin1 and EVI1, whereas miR-199a-5p and miR-214 accelerate senescence by targeting SIRT1 and Quaking [30,31,32,33,34,35]. Key modulators, miR-200c, miR-181b-5p, miR-30a-3p and miR-143, regulate proliferation, migration and calcific remodeling through KLF4/SUMO, HMGB1, ROCK2, and AKT pathways [36,37,38,39]. Moreover, exosomal miR-106a-5p (delivered via circHIPK3 under hyperglycemia) drives maladaptive EC–VSMC crosstalk, and loss of miR-542-3p unleashes BMP7-driven osteogenic trans differentiation [39,40]. Together, these miRNA networks integrate redox imbalance, chronic inflammation, cell-cycle arrest, and disrupted intercellular communication to promote arterial stiffening and impaired vascular regeneration. The resulting increase in systemic vascular resistance and microvascular dysfunction imposes a persistent afterload on the left ventricle, fosters adverse myocardial remodeling (hypertrophy, interstitial fibrosis), and impairs diastolic relaxation, hallmarks of HFpEF. In relation to the lymphatic dysfunction observed in aging hearts [20], miR-31-5p appears to block the endothelial-to-mesenchymal transition and fibrosis in LECs observed in various forms of HF [41].

### 4.2. LncRNA

**Figure 2:**
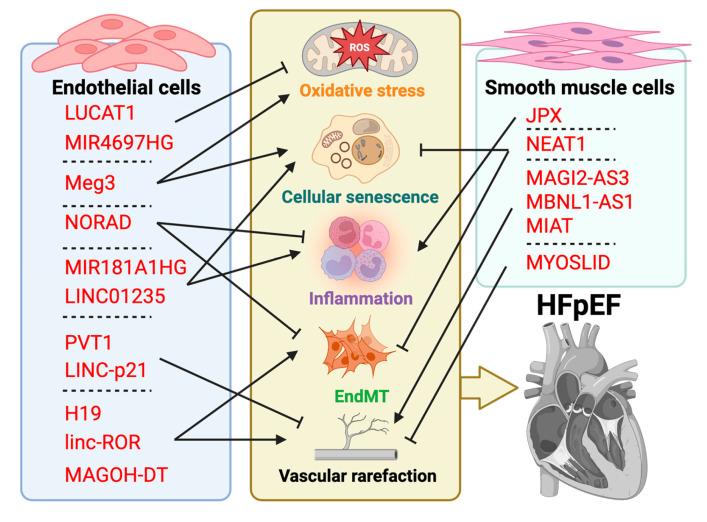
The role of lncRNAs in endothelial cells and smooth muscle cells in vascular aging and HFpEF. Created in Biorender. Chen, J. (2025) https://BioRender.com/4r9y7pe.

Long noncoding RNAs (lncRNAs) have emerged as versatile arbiters of EC senescence and vascular aging, exerting their effects via miRNA sponging, epigenetic remodeling and direct RNA–protein interactions to calibrate oxidative stress responses, inflammation, apoptosis, autophagy and endothelial–mesenchymal transition (EndMT) (Table 3 and Figure 2). For instance, LUCAT1 and MIR4697HG safeguard EC integrity under oxidized LDL stress by sequestering miR-6776-5p and engaging the FUS/ANXA5 axis to bolster autophagy, whereas Meg3 exacerbates mitochondrial dysfunction and p21/p16-driven growth arrest [79,94]. NORAD maintains quiescent endothelium by binding SFPQ to repress IL-8 transcription and blunt NF-κB/p53-p21 signaling, and RAMP2-AS1 enhances RAMP2 expression to similarly attenuate proinflammatory gene activation [81,95,96,97]. Conversely, MIR181A1HG and LINC01235 aggravate inflammasome activation and senescence via NLRP3 and miR-224-3p sponging, while protective lncRNAs such as MALAT1 and PRKAG2-AS1 mitigate monocyte adhesion and cytokine release through ATG5-dependent autophagy and PRKAG2 stabilization [72,85,92,98,99,100]. Finally, PVT1 and LINC-p21 directly counteract apoptosis and cell-cycle arrest through the miR-532-3p/MAPK1 and PI3K/AKT/mTOR axes, and H19, linc-ROR and MAGOH-DT choreograph EndMT and reparative neovascularisation via miR-107/FADD, miR-145/Smad3 and TGF-β2 signals, respectively [77,78,93,101,102,103,104]. Collectively, dysregulation of these lncRNA networks drives endothelial dysfunction and microvascular rarefaction, thereby heightening peripheral resistance, exacerbating left ventricular afterload and fueling the diastolic impairment that typifies HFpEF. In VSMCs, lncRNAs similarly dictate phenotypic switching, inflammation, calcification and cellular aging through competing endogenous RNA (ceRNA) axes and chromatin-level control (Table 4 and Figure 2). MAGI2-AS3, MBNL1-AS1 and MIAT promote pathological VSMC proliferation and migration by sponging miR-525-5p, miR-424-5p and miR-326 to activate MAPK, PI3K-Akt and EGR1–ELK1–ERK/KLF4 pathways, whereas the protective MYOSLID restrains Ang II-driven growth via miR-29c-3p [105,106,107,108,109]. The enhancer-like JPX lncRNA activates SASP by recruiting p65/BRD4 and triggering VDAC1-mediated DNA leakage with downstream STING activation, while NEAT1 guides EZH2 to silence senescent genes (p16, p21, TIMP3), promoting osteogenic trans differentiation and arterial stiffening [110,111].

### 4.3. CircRNA

**Figure 3:**
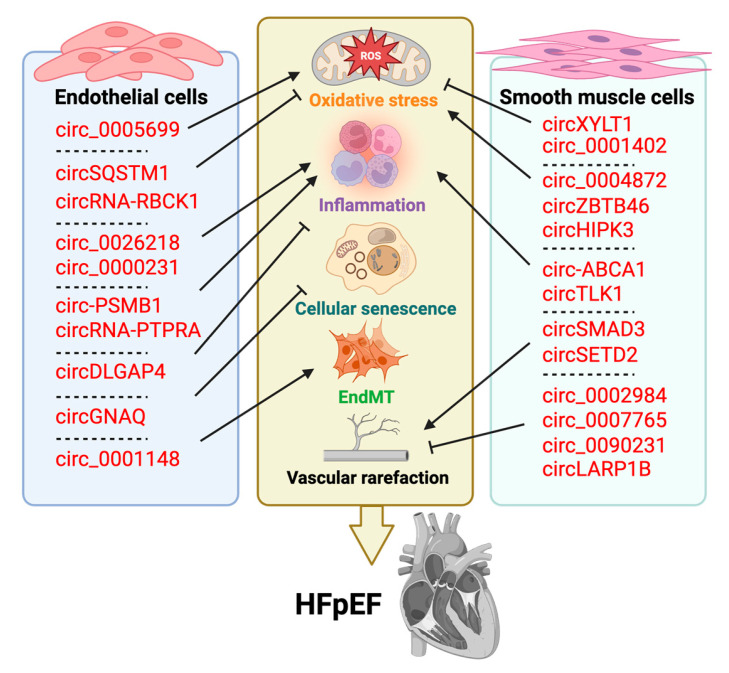
The role of circRNAs in endothelial cells and smooth muscle cells in vascular aging and HFpEF. Created in Biorender. Chen, J. (2025) https://BioRender.com/pz7k8d5.

Circular RNAs (circRNAs) are exceptionally stable, covalently closed loops that regulate EC function and senescence by sponging miRNAs, modulating RNA-binding proteins, and even encoding small regulatory peptides (Table 5 and Figure 3). In aging endothelium, several circRNAs exacerbate oxidative stress and inflammatory signaling. For example, circ_0005699 aggravates ROS accumulation and dysfunction by sequestering miR-384 to derepress ASPH [116], circ_0026218 amplifies NF-κB-driven inflammation via the miR-188-3p/TLR4 axis, and circ_0000231 promotes EC apoptosis through miR-590-5p/TXNIP-mediated NF-κB activation [112,117]. Conversely, protective circRNAs such as circSQSTM1 restore Sirt1 levels to attenuate oxidative damage and autophagic impairment [132], and statin-induced circRNA-RBCK1 preserves nitric oxide signaling by targeting miR-133a [144]. Inflammasome-associated circ-PSMB1 and circRNA-PTPRA further drive pyroptosis through ASC and miR-671-5p pathways [126,130], whereas circDLGAP4 limits inflammation and apoptosis by promoting autophagy via miR-134-5p/PTPN4 [123]. Finally, senescence-linked circGNAQ delays EC aging by inhibiting miR-146a-5p to restore PLK2 [124]. Circ_0001148 and related circRNAs trigger EndMT and maladaptive vascular remodeling via miR-218-5p/JMY [113]. In VSMCs, circRNAs orchestrate phenotypic switching, oxidative stress responses, inflammation, and senescence predominantly through miRNA sponging and RNA-binding protein interactions (Table 6 and Figure 3). CircSMAD3 and the circSETD2 encoded p-414aa peptide both suppress VSMC proliferation and neointima formation by stabilizing p53γ signaling or disrupting HuR/C-FOS interactions [145,146], whereas pro-remodeling circ_0002984, circ_0007765, circ_0090231 and circLARP1B promote VSMC growth, migration and synthetic transformation via let-7a-5p/KLF5, miR-654-3p/FRS2, miR-942-5p/PPM1B and cAMP/PDE4C axes, respectively [130,147,148,149]. Under oxidative stress, circXYLT1 protects against ROS through PTBP1-dependent chemokine regulation [150], but circ_0004872, circZBTB46, and circHIPK3 enhance mitochondrial DRP1-driven fragmentation, ROS accumulation, and necroptosis, exacerbating plaque vulnerability [134,151,152]. Inflammation-linked circ-ABCA1 and circTLK1 drive VSMC phenotypic switching via ROCK2 and KLF4 modulation [153,154], while circ_0001402 counteracts neointimal hyperplasia by enhancing autophagy through the miR-183-5p/FKBPL/BECN1 pathway [154]. By integrating metabolic, oxidative, and inflammatory cues, these circRNA-mediated networks dictate VSMC fate and arterial remodeling, driving increased PWV and loss of Windkessel function.

### 4.4. PiRNA

PIWI-interacting RNAs (piRNAs) are a class of 24–31-nucleotide small ncRNAs that associate with Piwi-clade Argonaute proteins to transcriptionally and post-transcriptionally silence transposable elements (TEs) and preserve genomic integrity. Although historically regarded as germline-specific, recent invertebrate studies demonstrate that piRNA levels and Piwi activity decline with age in somatic tissues [171]. C. elegans exhibits global drops in piRNA abundance over lifespan [172], and Drosophila overexpression of Piwi in intestinal stem cells mitigates age-related apoptosis, TE reactivation, and barrier dysfunction [173]. Intriguingly, germline-derived piRNAs have been shown to transcriptionally regulate Hedgehog-like ligands (with respect to-1/10), establishing a tunable germline-to-soma pro-aging signal that adjusts somatic resource allocation in response to reproductive cues. Beyond TE suppression, piRNA–Piwi complexes appear to modulate oxidative stress responses and inflammatory pathways via epigenetic remodeling, and their biogenesis is sensitive to posttranslational regulation—casein kinase II phosphorylation of USTC component TOFU-4, for example, is required for piRNA production and declines with age, linking piRNA dysfunction directly to somatic aging [174]. In the vasculature, loss of piRNA-mediated TE repression in ECs accelerates genomic instability and SASP activation, while epigenetic alterations orchestrated by dysregulated Piwi complexes dysregulate eNOS expression or adhesion molecule profiles [173]. In VSMCs, abnormal piRNA/TLR4 signaling exacerbates osteogenic transdifferentiation and arterial calcification [175]. Mechanistically, such vascular piRNA defects are likely to compound arterial stiffening, elevating PWV and left ventricular afterload, while impairing microvascular endothelial function and coronary perfusion reserve. These changes synergistically drive maladaptive myocardial remodeling, interstitial fibrosis, and diastolic dysfunction, characteristic features of HFpEF. Systematic profiling of piRNA expression in aging human and animal vessels, coupled with targeted ablation or overexpression studies in vascular cell types, will be essential to validate piRNAs as novel epigenetic regulators of vascular aging and as interventional targets to forestall HFpEF progression.

### 4.5. TsRNA

tRNA-derived small RNAs (tsRNAs or tRFs) form a diverse class of 15–40 nucleotide ncRNAs generated by precise cleavage of precursor or mature tRNAs. Once dismissed as mere degradation intermediates, tsRNAs are now recognized as stress-responsive regulators of gene expression at multiple levels: they can inhibit translation initiation by displacing eIF4F complexes, promote mRNA decay through Argonaute-dependent mechanisms, modulate epigenetic landscapes, and even act as paracrine signals when shuttled in extracellular vesicles. Their biogenesis is tightly controlled by ribonucleases such as angiogenin and Dicer, and is markedly upregulated under conditions of oxidative stress, nutrient deprivation, or ischemia, stressors intrinsically linked to the aging process. Consequently, tsRNAs accumulate in an age-dependent manner across species: Glu-5′-tsRNA-CTC rises in aged mammalian neurons where it impairs mitochondrial translation and cristae architecture, leading to synaptic glutamate deficits and memory decline. Additionally, Drosophila and C. elegans display global increases in specific tRFs with lifespan, suggesting conserved roles in systemic aging regulation. In the vasculature, tsRNAs have begun to emerge as critical mediators. Endothelial tsRNA-1599 is induced by angiogenic stress and binds YBX1 to repress HK2, thereby shifting metabolism away from glycolysis and exacerbating pathological neovascularization. Its inhibition restores glycolytic flux and attenuates aberrant vessel growth in diabetic retinopathy models [176]. Ischemia-generated tsRNAs similarly suppress endothelial repair programs, hinting at broader roles in modulating vascular integrity during aging. In VSMCs, exosome-delivered tsRNAs such as miR-106a-5p-like fragments can activate pro-survival and proliferative signaling, contributing to medial thickening and arterial stiffness [177]. Critically, circulating tsRNA signatures, most notably tRF-21-NB8PLML3E, have been linked to pathological cardiac hypertrophy, acting both as biomarkers and functional suppressors of cardiomyocyte growth [178]. By driving endothelial metabolic reprogramming, VSMC remodeling, and intercellular communication, tsRNAs thus represent a novel epigenetic axis bridging vascular aging to increased ventricular afterload, diastolic dysfunction, and HFpEF. Elucidating tsRNA-specific profiles and mechanisms in aged vessels and failing hearts promises to uncover innovative biomarkers and therapeutic targets for age-related cardiovascular disease.

### 4.6. SnRNA

Small nuclear RNAs (snRNAs), including U1, U2, U4, U5, and U6, are indispensable ncRNA components of the spliceosome, the macromolecular complex responsible for the precise excision of introns and ligation of exons from pre-mRNA transcripts [179]. This meticulous process of RNA splicing is fundamental for generating mature, functional mRNAs and ensuring proteome diversity and integrity. The fidelity and efficiency of pre-mRNA splicing are paramount for normal cellular function. However, accumulating evidence indicates that splicing regulation becomes progressively dysregulated with aging across various tissues. This age-associated decline in splicing fidelity can result from altered expression or modification of snRNAs themselves, changes in the abundance or activity of core spliceosomal proteins and auxiliary splicing factors [180], or an accumulation of oxidative damage-affected spliceosome components [181]. Such age-related splicing defects can have profound consequences on vascular aging. Dysregulated splicing can lead to the production of aberrant protein isoforms that impair vascular cell function by affecting endothelial barrier integrity, NO bioavailability, VSMC contractility, or proinflammatory signaling pathways. While direct, comprehensive studies extensively detailing the specific roles of individual snRNA species in orchestrating vascular aging are still relatively nascent, alterations in the expression of certain snRNAs or their post-transcriptional modifications (e.g., N6-methyladenosine modification of U6 snRNA, which has been linked to splicing regulation and cardiovascular health [182]) are plausible contributors to the observed age-related increase in aberrant splicing events within vascular cells. Furthermore, key splicing factors that interact with snRNAs to form functional small nuclear ribonucleoproteins (snRNPs), such as SRSF1 or PTBP1, have been shown to be dysregulated during cellular senescence and implicated in vascular dysfunction [183,184]. Thus, snRNA-mediated dysregulation of splicing in the aging vasculature could represent a significant, albeit underexplored, upstream mechanism contributing to HF development and progression. Moreover, it is conceivable that age-related splicing dysregulation, potentially involving snRNA alterations, also occurs directly within cardiomyocytes, impairing their contractile function, stress resilience, and promoting cardiac senescence and fibrosis. Further research is imperative to delineate the specific snRNA alterations and the landscape of aberrant splicing events that characterize vascular aging, to understand their mechanistic contributions to vascular cell dysfunction, and to clarify how these processes ultimately impact cardiac health and predispose to heart failure. Elucidating these pathways may unveil novel diagnostic markers or therapeutic targets aimed at preserving splicing fidelity to mitigate age-related cardiovascular decline.

### 4.7. SnoRNA

Small nucleolar RNAs (snoRNAs) are a highly conserved class of ncRNAs, typically 60–300 nucleotides in length, predominantly localized within the nucleolus, the primary site of ribosome biogenesis [185]. Canonically, snoRNAs function as guide molecules in the post-transcriptional modification of other ncRNAs, primarily ribosomal RNAs (rRNAs) and, in some cases, snRNAs and even mRNAs. They achieve this by forming small nucleolar ribonucleoproteins with a set of core proteins. The two major families, C/D box snoRNAs and H/ACA box snoRNAs, guide 2′-O-methylation and pseudouridylation, respectively, modifications crucial for ribosome assembly, stability, and translational fidelity [186]. Beyond these well-established roles, emerging evidence suggests that snoRNAs can also be processed into smaller, functional RNA species (snoRNA-derived RNAs, sdRNAs), act as miRNA precursors, or exert non-canonical functions in stress responses, alternative splicing, and gene expression regulation, hinting at a broader regulatory repertoire. The involvement of snoRNAs in vascular aging is a nascent but intriguing area of research. Given their central role in ribosome biogenesis and thus protein synthesis, dysregulation of snoRNA expression or function could profoundly impact vascular cell homeostasis during aging. Altered protein synthesis fidelity or capacity in aging ECs or VSMCs can contribute to cellular senescence, impaired stress responses, chronic inflammation, and aberrant ECM production, all hallmarks of vascular aging. SnoRNAs are also abundantly expressed in cardiomyocytes, where ribosome function and protein synthesis are paramount for maintaining cardiac structure, contractility, and adaptive responses to stress (e.g., hypertrophy). Thus, age-related alterations in snoRNA networks within the heart itself could directly impair cardiomyocyte proteostasis, promote cardiac senescence, fibrosis, and contribute to the failing phenotype [187]. Unraveling the specific snoRNAs and snoRNA-mediated pathways that are perturbed during vascular and cardiac aging, and understanding their contribution to HF, represents an important frontier.

Beyond the extensively characterized miRNAs, lncRNAs, circRNAs, piRNAs, snRNAs, snoRNAs, and tsRNAs, a growing body of evidence implicates additional ncRNA classes in the regulation of vascular aging and HF. Enhancer RNAs (eRNAs) orchestrate endothelial and smooth muscle gene expression through enhancer–promoter looping, while Y RNAs and their derived fragments propagate inflammatory and survival signals within the vascular wall [188,189]. Small Cajal body RNAs (scaRNAs) ensure proper snRNA maturation and spliceosomal function, thereby influencing alternative splicing programs in both VSMCs and cardiomyocytes, and vault RNAs modulate autophagy and stress-response pathways critical for vascular and cardiac cell homeostasis [190,191]. Collectively, these diverse noncoding RNAs govern transcriptional, post-transcriptional, and epigenetic mechanisms to drive endothelial dysfunction, arterial stiffening, and maladaptive myocardial remodeling, core features of age-related cardiovascular disease. Delineating their precise roles via integrated transcriptomic profiling, mechanistic studies, and in vivo validation will be essential to uncover novel biomarkers and therapeutic targets, ultimately enabling precision strategies to attenuate vascular aging and forestall HF.

## 5. Clinical Translation and Therapeutic Perspectives

### 5.1. Diagnostic and Prognostic Value of Circulating ncRNAs in Heart Failure

The quest for robust, noninvasive biomarkers in HFpEF has propelled circulating ncRNAs into the spotlight. MiRNAs and lncRNAs are secreted by ECs, cardiomyocytes, and fibroblasts within exosomes, microvesicles, or bound to lipoprotein complexes, enabling their stable detection in plasma, serum, and urine. Their disease-specific expression profiles mirror the molecular derangements of both vascular aging and myocardial remodeling, making them ideal candidates for early diagnosis, prognostication, and therapeutic monitoring.

MicroRNA as Biomarkers. Elevated plasma miR-21, a central mediator of fibrogenic pathways, correlates with cardiac collagen volume fraction and predicts declines in peak VO_2_ during exercise testing [192]. Similarly, miR-423-5p shows strong associations with left atrial volume and E/e’ ratio on echocardiography, reflecting adverse ventricular remodeling and elevated filling pressures. In contrast, circulating miR-126 tracks endothelial health—lower levels predict diminished flow-mediated dilation and higher incidence of HFpEF hospitalizations, while miR-126 supplementation in preclinical models restores angiogenesis and ameliorates diastolic stiffness [193].

lncRNA as Biomarkers. The lncRNA LIPCAR is unique for its prognostic independence from natriuretic peptides. Elevated LIPCAR in early convalescence after acute decompensation forecasts rehospitalization and mortality over a 2-year follow-up, even in patients with HFpEF [194]. Other lncRNAs, such as ANRIL and CHROME, have also emerged as correlates of coronary microvascular function and cardiomyocyte viability, offering potential for multi-marker panels [195].

Technological Advances and Clinical Implementation. Advances in digital droplet PCR and targeted RNA sequencing now permit absolute quantification of low-abundance ncRNAs with high specificity and reproducibility. Standardization of preanalytical protocols, encompassing blood collection, exosome isolation, and RNA extraction, has improved interlaboratory concordance, paving the way for large-scale validation studies. Furthermore, dynamic changes in circulating ncRNA levels have been shown to reflect responses to therapies such as SGLT2 inhibitors and soluble guanylate cyclase stimulators, suggesting utility in real-time monitoring of treatment efficacy [196].

Limitations and Future Directions. Clinical application faces challenges such as heterogeneous patient populations, comorbidities (e.g., renal dysfunction) that alter ncRNA clearance, and the need for age- and sex-specific reference ranges. Future multicenter trials should integrate ncRNA profiling with imaging and hemodynamic assessments to refine risk models and identify threshold values that trigger clinical intervention. As our understanding deepens, circulating ncRNAs are poised to become integral to precision cardiovascular medicine, enabling early detection of HFpEF, stratification of patient subphenotypes, and personalized therapeutic guidance.

### 5.2. Therapeutic Targeting of ncRNAs: ASOs, Mimics, and Inhibitors

Building upon their established roles in driving vascular inflammation, maladaptive remodeling, and diastolic dysfunction, ncRNAs have emerged as compelling therapeutic targets in HFpEF. Three principal modalities, antisense oligonucleotides (ASOs), miRNA mimics, and miRNA inhibitors (antagomiRs), have advanced to preclinical and early clinical evaluation, each with unique strengths and challenges.

Antisense Oligonucleotides (ASOs). ASOs are synthetic, single-stranded nucleic acids engineered to bind target ncRNAs via Watson–Crick base pairing, triggering RNase H–mediated degradation or steric blockade of function. In rodent models of vascular aging, ASOs targeting lncRNA MALAT1 restore endothelial NO production, reduce VCAM-1 expression, and improve microvascular perfusion [197]. Similarly, ASO-mediated knockdown of H19 in pressure-overload mice attenuates VSMC proliferation and arterial stiffening, translating to improved diastolic parameters [198]. Chemical modifications, such as locked nucleic acids (LNAs) and phosphorothioate backbones, enhance ASO stability and affinity, while reducing immunogenicity. However, achieving efficient delivery to the endothelium or myocardium remains a critical barrier. Systemically administered ASOs accumulate predominantly in the liver and kidney. Thus, cardiovascular applications increasingly rely on targeted delivery vehicles, such as polyethylene glycol–coated liposomes conjugated with peptides or antibodies that recognize endothelial surface markers (e.g., ICAM-1) to concentrate ASOs at sites of vascular injury.

miRNA Mimics. MiRNA mimics are double-stranded RNA duplexes designed to replenish downregulated protective miRNAs. Restoration of miR-126 via lipid nanoparticle–encapsulated mimics enhances endothelial progenitor cell mobilization, angiogenesis, and NO bioavailability in aged hypertensive rats, leading to improved myocardial perfusion and reduced interstitial fibrosis [199]. Tailored chemical modifications, 2′-O-methylation and partial phosphorothioate linkages, improve mimic stability and reduce off-target silencing. Local delivery approaches, such as intramyocardial injection during catheter-based interventions or incorporation into bioresorbable scaffolds, further increase tissue specificity while minimizing systemic exposure [200]. Nevertheless, systemic delivery of mimics carries risks of innate immune activation and unintended repression of non-cardiovascular targets, underscoring the need for rigorous biodistribution and safety profiling.

miRNA Inhibitors (AntagomiRs). AntagomiRs are single-stranded, chemically stabilized oligonucleotides that sequester overexpressed pathologic miRNAs. In preclinical HFpEF models, antagomiRs against miR-34a and miR-21 reduce endothelial senescence and myocardial fibrosis, respectively, by derepressing SIRT1 and PTEN pathways. Conjugation of antagomiRs to cholesterol or cell-penetrating peptides facilitates cellular uptake, but also increases nonspecific distribution. Advanced strategies employ aptamer–antagomiR chimeras, in which a vascular-targeting aptamer (e.g., against VCAM-1) directs the inhibitor to activated endothelium, achieving more selective inhibition with lower total doses.

Emerging Delivery Platforms. To overcome delivery hurdles, researchers are exploring innovative vectors that combine specificity, efficiency, and safety:

(1) Ligand-targeted nanoparticles functionalized with antibodies or peptides that recognize vascular cell markers (e.g., ICAM-1, E-selectin) enrich payloads in diseased vasculature.
(2) Exosome-based vehicles, leveraging the natural tropism of engineered exosomes carrying therapeutic ncRNAs, can home to injured myocardium and endothelium while evading immune surveillance.
(3) Virus-derived vectors (AAV, lentivirus) offer prolonged expression of miRNA sponges or decoy RNAs but require rigorous screening to mitigate insertional mutagenesis and immunogenicity.
(4) Cell-type–specific promoters incorporated into plasmid or viral constructs restrict ncRNA modulation to endothelial cells (e.g., using VE-cadherin promoter) or cardiomyocytes (e.g., α-MHC promoter), reducing off-target effects.

Challenges and Future Directions. Despite encouraging preclinical efficacy, clinical translation of ncRNA therapeutics must address several key issues: long-term safety in the context of chronic cardiovascular disease, potential for unintended gene network perturbations, and scalable manufacturing under good manufacturing practice conditions. Biomaterial-based controlled release systems, tunable by local enzymatic or pH cues, may offer spatiotemporal precision in ncRNA delivery. Moreover, integration of noninvasive imaging biomarkers, such as radiolabeled ASOs or PET-detectable nanoparticles, can enable real-time monitoring of target engagement and therapeutic distribution [201]. As the field advances, a synergistic combination of ncRNA therapeutics with existing pharmacotherapies (e.g., SGLT2 inhibitors, MR antagonists) holds promise for multifaceted intervention against the vascular–cardiac axis in HFpEF.

### 5.3. Personalized Therapies Informed by Multi-Omics and ncRNA Profiling

The heterogeneity of HFpEF, underpinned by distinct manifestations of vascular aging, arterial stiffness, microvascular rarefaction, and endothelial senescence, and modulated by ncRNAs, demands therapies tailored to each patient’s vascular–ncRNA endotype. Integrating circulating and tissue ncRNA profiles with vascular phenotyping (e.g., PWV, coronary flow reserve) and multi-omics layers (transcriptomics, proteomics, metabolomics, epigenomics) enables the stratification of HFpEF into subgroups driven predominantly by macrovascular stiffening, microvascular dysfunction, or inflammaging.

For patients with a “stiff arteries” endotype, characterized by elevated PWV and downregulated miR-29 in both plasma and arterial biopsies [9], combined therapy with miR-29 mimics (to repress pathologic collagen cross-linking) and agents that restore elastin integrity (e.g., AGE breakers) may directly reverse ECM accumulation in the aorta and myocardium. In contrast, the “microvascular deficit” endotype, identified by reduced miR-126 and lncRNA MALAT1 upregulation alongside diminished coronary flow reserve, could benefit from endothelial-targeted delivery of miR-126 mimics, enhancing capillary density and NO bioavailability, paired with NO–cGMP enhancers to restore perfusion reserve and diastolic relaxation. Patients exhibiting an “inflammaging” profile, marked by elevated exosomal miR-21, miR-217, and increased SASP-associated lncRNAs (e.g., ANRIL) in concert with high-sensitivity coronary flow reserve and IL-6, may derive maximal benefit from antagomiRs against these proinflammatory ncRNAs, in combination with IL-6 receptor antagonists or SGLT2 inhibitors that blunt systemic inflammation and oxidative stress.

Advanced machine-learning models can integrate serial liquid-biopsy ncRNA measurements, wearable-device hemodynamics, and imaging biomarkers (e.g., myocardial extracellular volume), dynamically updating risk scores and predicting which vascular–ncRNA modulator will most effectively restore vascular compliance, microvascular health, and myocardial relaxation. By aligning ncRNA-directed interventions with each patient’s specific vascular aging phenotype, this precision-medicine framework moves beyond symptomatic management to targeted, mechanism-based reversal of the vascular–cardiac aging axis in HFpEF.

## 6. Conclusions and Future Perspectives

HFpEF is a common and complex form of HF, especially in older adults, with vascular aging playing a central role in its pathophysiology. Over the past decade, ncRNAs have emerged as key regulators linking vascular aging to cardiac dysfunction, significantly advancing our understanding of HFpEF and presenting new therapeutic avenues. Nevertheless, translating these insights into clinical practice remains challenging due to limited human validation and complex disease heterogeneity.

Despite significant progress, substantial gaps and controversies remain. Most ncRNA studies in HFpEF are limited to preclinical models or isolated cellular pathways, and clear causal relationships between ncRNA dysregulation and HFpEF progression have not been definitively confirmed in clinical studies. The pronounced clinical heterogeneity of HFpEF presents significant challenges in identifying universally applicable ncRNA biomarkers or therapeutic targets. Moreover, translating ncRNA findings into clinical practice is further complicated by variability in patient populations, disease etiologies, and coexisting conditions. Additionally, the role of ncRNAs in lymphatic function remains largely unexplored in HFpEF, although preliminary data from other cardiovascular contexts suggest important regulatory functions in lymphangiogenesis and lymphatic endothelial integrity. Addressing these controversies and expanding ncRNA studies into clinical validation will be crucial for advancing translational potential.

To overcome these translational hurdles, future research must integrate mechanistic studies, rigorous clinical validation, and comprehensive patient phenotyping across diverse populations. Emerging technologies such as single-cell RNA sequencing and spatial transcriptomics are enhancing our understanding of ncRNA dynamics at unprecedented resolution, revealing critical interactions within aging cardiovascular systems. Integration with multi-omics approaches, including epigenomics, proteomics, and metabolomics, will facilitate the mapping of complex ncRNA regulatory networks and the identification of potential therapeutic targets. Despite the promising translational potential of RNA-targeted therapies, including ASOs, miRNA mimics, and CRISPR-based RNA editing, significant challenges remain regarding effective delivery, specificity, and long-term safety profiles. Addressing these issues will be pivotal in moving ncRNA-based therapies from experimental stages to clinical application.

Looking forward, ncRNA-based biomarkers and therapeutics hold significant promise for transforming the diagnosis, stratification, and management of HFpEF. Circulating ncRNAs, in particular, offer noninvasive biomarkers for disease monitoring, patient stratification, and prediction of therapeutic response. Integrating ncRNA profiling into clinical trials and therapeutic algorithms can potentially facilitate personalized and mechanism-driven care for HFpEF patients. Ultimately, combining insights from vascular aging biology, lymphatic function, ncRNA regulation, and comprehensive systems-level analyses presents a paradigm shift in HFpEF management, redefining it as a mechanistically tractable condition rather than an untreatable syndrome. Achieving this vision requires sustained multidisciplinary research and substantial translational investments.

## Figures and Tables

**Table 1:** Regulatory Functions of miRNAs in Endothelial Cells in Vascular Aging.

miRNA	Regulatory Mechanisms	Target(s)	
miR-217	miR-217 inhibits eNOS and upstream VEGF/Apelin signaling, contributing to endothelial dysfunction and atherosclerosis.	eNOS	[42]
miR-126	miR-126 maintains HIF-1α levels, supporting endothelial angiogenesis and repair; its downregulation impairs function.	HIF-1α	[43]
miR-181b	Butyrate activates PPARδ to upregulate miR-181b, suppressing NOX2-mediated ROS and improving endothelial function.	NOX2	[44]
miR-1-3p	AGEs downregulate miR-1-3p, activating the MLCK/p-MLC pathway to induce endothelial senescence and barrier dysfunction.	MLCK; p-MLC	[45]
miR-128	MEG3 inhibits miR-128 to upregulate Girdin, promoting antioxidation and delaying endothelial senescence.	Girdin	[46]
miR-34b	IDH2 deficiency upregulates miR-34b/c, inhibiting mitophagy and reducing Sirt3, inducing ROS accumulation and senescence.	Sirt3	[47]
miR-200c	ROS induces miR-200c, which inhibits ZEB1 and promotes endothelial growth arrest, apoptosis, and senescence.	ZEB1	[48]
miR-4500	Oxidative stress induces OIP5-AS1, which inhibits miR-4500 and upregulates ARG2, promoting endothelial dysfunction.	ARG2	[49]
miR-29	TGF-β/Smad signaling upregulates miR-29a/c, suppressing Suv4-20h and impairing DNA repair to drive senescence.	Suv4-20h	[50]
miR-1	Hepatocyte-derived EVs carrying miR-1 suppress KLF4 and activate NF-κB, inducing endothelial inflammation and atherosclerosis.	KLF4	[51]
miR-216a	miR-216a promotes endothelial senescence and inflammation by targeting Smad3 and destabilizing IκBα.	Smad3	[52]
miR-34a-5p	miR-34a-5p activates p53 and p16 pathways to drive endothelial inflammation and senescence.	p53; p16	[53]
miR-181b-5p	Low shear stress downregulates miR-181b-5p, relieving suppression of STAT3 and activating NLRP3 inflammasome.	STAT3	[54]
miR-34a	Kallistatin upregulates Let-7g and suppresses miR-34a to enhance SIRT1/eNOS and antioxidant enzymes.	SIRT1; eNOS	[55]
miR-27a-3p	miR-27a-3p suppresses GRIN2D and the PKC/MEK/ERK pathway to alleviate endothelial oxidative stress and senescence.	GRIN2D	[56]
miR-25-3p	miR-25-3p targets TULA-2 to activate SYK/VEGFR-2 phosphorylation and improve angiogenesis in aging.	SYK; VEGFR-2	[57]
miR-217	miR-21 and miR-217 in EVs suppress DNMT1 and SIRT1 in recipient cells to induce senescence via paracrine signaling.	DNMT1; SIRT1	[26]
miR-181b	miR-181b targets MAP3K3 to inhibit MAPK signaling and reduce oxidative stress-induced endothelial apoptosis.	MAP3K3	[58]
miR-217	miR-217 suppresses SIRT1 and activates p53, impairing endothelial proliferation and angiogenesis.	SIRT1	[59]
miR-217	miR-217 inhibits VEGF/apelin/eNOS signaling, impairing NO generation and promoting dysfunction.	eNOS; VEGF	[42]
miR-155-5p	KRGE induces HO-1 and suppresses NF-κB/miR-155-5p to restore eNOS and delay senescence.	eNOS	[60]
miR-10A	miR-10A and miR-21 suppress HMGA2, activating p16/p19 to induce EPC senescence and impair repair.	HMGA2	[61]
miR-409	miR-409 suppresses CCL5/VEGF signaling in EPCs, reducing angiogenesis and promoting senescence.	VEGF	[62]
Hsa-miR-409-3p	miR-409-3p targets PPP2CA to activate p38/JNK and impair EPC angiogenesis, promoting senescence.	PPP2CA	[63]
miR-15b-5p	MALAT1 sponges miR-15b-5p to upregulate MAPK1 and activate mTOR, suppressing EPC autophagy and increasing inflammation.	MAPK1	[64]
miR-361-5p	miR-361-5p targets cytoskeletal/ECM genes; restoration reduces senescence but requires precise control.		[65]
miR-146a	MSC-sEV-delivered miR-146a suppresses Src kinase and stabilizes VE-cadherin, reducing inflammation and restoring angiogenesis.	Src	[66]
miR-145	miR-145 suppresses Sema3a to maintain the neurovascular interface; its downregulation in aging impairs innervation and function.	Sema3a	[67]
miR1204	miR-1204 downregulates MYLK, promoting SASP and inflammatory phenotype in VSMCs, exacerbating vascular aging.	MYLK	[68]
miR-34a-5p	miR-34a-5p suppresses MARCHF8, increasing ADAM10, leading to endothelial dysfunction and senescence.	MARCHF8	[69]
miR-199a-3p	High glucose downregulates miR-199a-3p, relieving DDR1 inhibition and activating p53/p21 to induce senescence.	DDR1	[70]
miR-214	miR-214 in EC-derived exosomes suppresses ATM in recipient cells to reduce senescence and promote angiogenesis.	ATM	[71]

Abbreviations: eNOS: endothelial nitric oxide synthase; VEGF: vascular endothelial growth factor; PPARδ: peroxisome proliferator-activated receptor delta; NOX2: NADPH oxidase 2; ROS: reactive oxygen species; MLCK: myosin light-chain kinase; p-MLC: phosphorylated myosin light-chain; IDH2: isocitrate dehydrogenase 2; SIRT1/3: sirtuins; ZEB1: zinc finger E-box-binding homeobox 1; ARG2: arginase 2; EVs: extracellular vesicles; DNMT1: DNA methyltransferase 1; Smad3: SMAD family member 3; IκBα: NF-κB inhibitor alpha; TULA-2: T-cell ubiquitin ligand-2; SYK: spleen tyrosine kinase; EPC: endothelial progenitor cell; MAPK: mitogen-activated protein kinase; MAP3K3: MAPK kinase kinase 3; PKC: protein kinase C; MEK: MAPK/ERK kinase; ERK: extracellular signal-regulated kinase; THAP1: THAP domain containing protein 1; ATM: ataxia telangiectasia mutated; ECM: extracellular matrix; NF-κB: nuclear factor kappa-light-chain-enhancer of activated B cells; SASP: senescence-associated secretory phenotype.

**Table 2:** Regulatory Functions of lncRNAs in Endothelial Cell Senescence and Vascular Aging.

LncRNA	Regulatory Mechanisms	Target(s)	
LINC01235	Inhibits autophagy via miR-224-3p/RABEP1	miR-224-3p; RABEP1	[72]
TCONS_02443383	Activates PPAR and adhesion genes	PPAR	[73]
uc003pxg.1	Enhances TGF-β1/α-SMA via miR-339-5p	miR-339-5; TGF-β1; α-SMA	[74]
NORAD	Promotes EC proliferation, suppresses ferroptosis via miR-106a/CCND1	miR-106a; CCND1	[75]
linc-ROR	Promotes EndMT via miR-145/Smad3 axis	miR-145; Smad3	[76]
MAGOH-DT	Enhances TGF-β2 translation via HNRPC binding	HNRPC; TGF-β2	[77]
PVT1	Promotes EC repair via miR-532-3p/MAPK1	miR-532-3p; MAPK1	[78]
LUCAT1	Resists oxidative stress via miR-6776-5p/LRRC25	miR-6776-5p; LRRC25	[79]
Meg3	Induces EC senescence via p21/p16 and mitochondrial dysfunction	p21; p16	[80]
NORAD	Blocks IL-8/NF-κB/p53-p21 to inhibit apoptosis and senescence	IL-8; NF-κB; p53; p21	[81]
MIR181A1HG	Activates NLRP3 inflammasome via Foxp1/NF-κB	Foxp1; NF-κB/p65; NLRP3	[82]
H19	Stabilizes H19 via METTL3-mediated m6A, promotes pyroptosis	IGF2BP2	[83]
SMANTIS	Modulates RUNX1 binding via Alu elements	RUNX1; EP300/CBFB	[84]
MALAT1	Promotes autophagy, suppresses adhesion via miR-30b-5p/ATG5	miR-30b-5p; ATG5	[85]
MIR4697HG	Inhibits EC damage via FUS/ANXA5 axis	FUS; ANXA5	[86]
MALAT1	Regulates VSMC and macrophage behavior in AS	EGR1-ELK1-ERK; KLF4	[87]
LASER	Inhibits LDL clearance via PCSK9/LDLR suppression	PCSK9; PCSK9	[88]
H19	Blocks STAT3 signaling to prevent EC senescence	STAT3	[89]
RAMP2-AS1	Cis-activation of RAMP2 and IL-8 regulation via SFPQ	ADM-RAMP2	[90]
lincRNA-p21	Inhibits autophagy, stabilizes junctions via PI3K/AKT/mTOR	PI3K/AKT/mTOR; miR-101-3p	[91]
PRKAG2-AS1	Activates PRKAG2, suppresses inflammation and apoptosis	PRKAG2	[92]
H19	Promotes EPC regeneration via miR-107/FADD, inhibits pyroptosis	miR-107; FADD	[93]

Abbreviations: EPC: endothelial progenitor cell; NF-κB: nuclear factor kappa-light-chain-enhancer of activated B cells; SASP: senescence-associated secretory phenotype; ROS: reactive oxygen species; PI3K/AKT/mTOR: phosphoinositide 3-kinase/protein kinase B/mammalian target of rapamycin; TGF-β: transforming growth factor beta; MAPK: mitogen-activated protein kinase; STAT3: signal transducer and activator of transcription 3; FADD: Fas-associated protein with death domain; PRC2: polycomb repressive complex 2; CDKN2A/B: cyclin-dependent kinase inhibitor 2A/B; EZH2: enhancer of zeste homolog 2; SFPQ: splicing factor proline and glutamine rich; ATG5: autophagy related 5; GPX4: glutathione peroxidase 4; DDR1: discoidin domain receptor 1; EGR1: early growth response 1; VCAM-1: vascular/intercellular cell adhesion molecule 1.

**Table 3:** Regulatory Functions of circRNAs in Endothelial Cell Senescence and Vascular Aging.

CircRNA	Regulatory Mechanisms	Target(s)	
Circ_0000231	Sponges miR-590-5p to upregulate TXNIP and activate NF-κB signaling	TXNIP; NF-κB	[112]
Circ_0001148	Sponges miR-218-5p to upregulate JMY and promote EndMT	miR-218-5p; JMY	[113]
Circ_0003204	Increases E-cadherin and HDAC9 by sponging miR-942-5p	miR-942-5p; HDAC9	[114]
Circ_0005699	Regulates the miR-450b-5P/NFKB1 axis	miR-450b-5P; NFKB1	[115]
Circ_0005699	Sponges miR-384 to upregulate ASPH and promote ox-LDL-induced EC injury	miR-384; ASPH	[116]
Circ_0026218	Sponges miR-188-3p to upregulate TLR4, activates NF-κB and propagates via exosomes	miR-188-3p; TLR4	[117]
Circ_0074673	Regulates the miR-1200/MEOX2 axis	miR-1200; MEOX2	[118]
Circ_0086296	Forms circ_0086296/miR-576-3p/IFIT1/STAT1 feedback loop	miR-576-3p; IFIT1; STAT1	[119]
Circ-AFF1	Regulates the miR-516b/SAV1/YAP1 axis	miR-516b; SAV1; YAP1	[120]
Circ-CHMP5	Sponges miR-532-5p to upregulate ROCK2 and promote EC injury	miR-532-5p; ROCK2	[121]
Circ-COL1A2	Regulates the miR-29b/VEGF axis	miR-29b; VEGF	[122]
Circ-DLGAP4	Sponges miR-134-5p to upregulate PTPN4 and induce autophagy, reduce apoptosis/inflammation	miR-134-5p; PTPN4	[123]
Circ-GNAQ	Sponges miR-146a-5p to upregulate PLK2 and delay EC senescence	miR-146a-5p; PLK2	[124]
Circ-GSE1	Regulates the miR-323-5p/NRP1 axis	miR-323-5p; NRP1	[125]
Circ-PSMB1	Sponges miR-624-3p to upregulate ASC and activate NLRP3 inflammasome	miR-624-3p; NLRP3	[126]
Circ-PVT1	Regulates the miR-24-3p/CDK4/pRb pathway	miR-24-3p; CDK4; pRb	[127]
Circ-RBCK1	Sponges miR-133a to block pathogenic targeting under statin stimulation	miR-133a	[128]
CircRNA-LONP2	Sponges miR-200a-3p to upregulate Keap1/YAP1/EZH2, suppress Nrf2/HO-1, promote stress/inflammation	miR-200a-3p; Keap1; YAP1	[129]
CircRNA-PTPRA	Sponges miR-671-5p to modulate inflammation and apoptosis	miR-671-5p	[130]
Circ-RSF1	Regulates the miR-758/CCND2 and miR-135b-5p/HDAC1 axes	miR-758; CCND2	[131]
Circ-SQSTM1	Sponges miR-23b-3p to upregulate Sirt1; promotes FOXO1 mRNA export and Sirt1 transcription	miR-23b-3p; Sirt1	[132]
Circ-USP36	Sponges miR-98-5p to elevate VCAM1; sponges miR-637 to enhance WNT4	miR-98-5p; VCAM1	[133]
Circ-ZBTB46	Stabilizes hnRNPA2B1, activates PTEN/AKT/mTOR, enhances proliferation/migration, inhibits apoptosis	hnRNPA2B1	[134]

Abbreviations: ASC: apoptosis-associated speck-like protein containing a CARD; ASPH: aspartate β-hydroxylase; CCND2: cyclin D2; CDK4: cyclin-dependent kinase 4; EZH2: enhancer of zeste homolog 2; FOXO1: forkhead box O1; HDAC1/9: histone deacetylase 1/9; HO-1: heme oxygenase 1; Keap1: Kelch-like ECH-associated protein 1; MEOX2: mesenchyme homeobox 2; NF-κB: nuclear factor kappa-light-chain-enhancer of activated B cells; NRP1: neuropilin-1; PLK2: polo-like kinase 2; PTEN: phosphatase and tensin homolog; PTPN4: protein tyrosine phosphatase, non-receptor type 4; ROCK2: Rho-associated coiled-coil containing protein kinase 2; SAV1: Salvador homolog 1; SIRT1: sirtuin 1; STAT1: signal transducer and activator of transcription 1; TLR4: Toll-like receptor 4; TXNIP: thioredoxin-interacting protein; VEGF: vascular endothelial growth factor; VCAM1: vascular cell adhesion molecule 1; WNT4: Wnt family member 4); YAP1: Yes-associated protein 1.

**Table 4:** Regulatory Functions of miRNAs in Vascular Smooth Muscle Cell Senescence and Vascular Aging.

miRNA	Regulatory Mechanisms	Target(s)	
miR-106a-5p	circHIPK3-enriched exosomes from ECs promote VSMC proliferation and inhibit apoptosis via miR-106a-5p/Foxo1/Vcam1 axis.	Foxo1; Vcam1	[135]
miR-199a-5p	miR-199a-5p upregulation promotes ROS by repressing Sirt1, exacerbating Ang II-induced VSMC senescence in AAA.	Sirt1	[32]
miR-200c	miR-200c disrupts SUMOylated KLF4 feedback loop by inhibiting Ubc9 and KLF4, suppressing VSMC proliferation and remodeling.	Ubc9; KLF4	[136]
miR-214	miR-214 suppresses Quaking to impair angiogenesis and promote VSMC senescence, serving as a vascular aging biomarker.	Quaking	[137]
miR-30a-3p	miR-30a-3p targets ROCK2 to suppress VSMC proliferation, migration, and phenotypic switching in ASO.	ROCK2	[138]
miR-22	Palmitic acid upregulates miR-22 to inhibit EVI1, preventing VSMC synthetic phenotype switching.	EVI1	[34]
miR-181b-5p	miR-181b-5p downregulation derepresses HMGB1, driving VSMC phenotype switching and vascular remodeling in hypertension.	HMGB1	[139]
miR-143	MEF2A induces miR-143 to inhibit AKT pathway, promoting H2O2-induced VSMC senescence.	AKT	[140]
miR-542-3p	miR-542-3p targets BMP7 to suppress osteogenic differentiation; its downregulation promotes VSMC calcification in aging.	BMP7	[141]
miR-30a	Rapamycin downregulates miR-30a to activate Beclin1-mediated autophagy and inhibit VSMC senescence.	Beclin1	[142]
miR-665	GAS5 sponges miR-665 to derepress SDC1, delaying VSMC senescence and protecting against vascular aging.	SDC1	[143]

Abbreviations: AKT: protein kinase B; ASO: arteriosclerosis obliterans; BMP7: bone morphogenetic protein 7; ECs: endothelial cells; EVI1: ecotropic viral integration site 1; FOXO1: forkhead box O1; HMGB1: high mobility group box 1; KLF4: Krüppel-like factor 4; MEF2A: myocyte enhancer factor 2A; mTOR: mechanistic target of rapamycin; Quaking: RNA-binding protein; ROCK2: Rho-associated protein kinase 2; ROS: reactive oxygen species; SDC1: syndecan-1; SIRT1: sirtuin 1; SUMO: small ubiquitin-like modifier; Ubc9: ubiquitin-conjugating enzyme E2I; Vcam1: vascular cell adhesion molecule 1; VSMC: vascular smooth muscle cell.

**Table 5:** Regulatory Functions of lncRNAs in Vascular Smooth Muscle Cell Remodeling and Aging.

LncRNA	Regulatory Mechanisms	Target(s)	
MAGI2-AS3	Sponges miR-525-5p to promote pathological VSMC proliferation and migration	miR-525-5p	[155]
MBNL1-AS1	Sponges miR-424-5p to modulate MAPK/PI3K-Akt signaling	miR-424-5p; MAPK; PI3K-Akt	[156]
MYOSLID	Sponges miR-29c-3p to suppress Ang II-induced VSMC migration, proliferation, and apoptosis	miR-29c-3p	[109]
MIAT	Sponges miR-326 to enhance MCP-1 expression and VSMC migration	miR-326; MCP-1	[157]
JPX	Enhancer-like RNA that activates SASP via chromatin remodeling and the STING pathway	SASP; VDAC1	[110]
NEAT1	Guides EZH2 to repress P16/P21/TIMP3 via histone methylation	EZH2; P16, P21 and TIMP3	[111]
SMILR	Acts in cis to scaffold chromatin and promote HAS2 transcription	CENPF	[158]
MIAT	Controls proliferation, apoptosis, and phenotypic switching in advanced AS	EGR1-ELK1-ERK; KLF4	[159]

Abbreviations: VSMC: vascular smooth muscle cell; SASP: senescence-associated secretory phenotype; EZH2: enhancer of zeste homolog 2; MCP-1: monocyte chemoattractant protein 1; HAS2: hyaluronan synthase 2; AS: atherosclerosis.

**Table 6:** Regulatory Functions of circRNAs in Vascular Smooth Muscle Cell (VSMC) Cell Senescence and Vascular Aging.

CircRNA	Regulatory Mechanisms	Target(s)	
Circ-NRG-1	miR-193b-5p/NRG-1 signaling modulation	miR-193b-5p; NRG-1	[160]
Circ-MAP3K5	miR-22-3p/TET2 axis regulation	miR-22-3p; TET2	[161]
Circ-ACTA2	NF-κB/NLRP3 pathway activation	NF-κB; NLRP3	[162]
Circ-SETD2(14,15)	HuR/C-FOS axis regulation by circSETD2(14,15)-encoded protein	p-414aa; HuR; C-FOS	[145]
Circ-HIPK3	DRP1/ROS-mediated mitochondrial fragmentation	DRP1	[163]
Circ-XYLT1	PTBP1-dependent chemokine signaling modulation	PTBP1	[150]
Circ-Esyt2	PCBP1-mediated p53β splicing regulation	p53β; PCBP1	[164]
Circ-Lrp6	miR-145 repression	miR-145	[165]
Circ-TEX14	miR-6509-3p/THAP1 pathway regulation	miR-6509-3p; THAP1	[166]
Circ-Diaph3	IGF-1 pathway activation via miR-148a-5p inhibition	miR-148a-5p; IGF1	[167]
Circ-ZXDC	miR-125a-3p/ABCC6 signaling regulation	miR-125a-3p; ABCC6	[168]
Circ-CHFR	miR-370/FOXO1/Cyclin D1 modulation	miR-370; FOXO1; Cyclin D1	[169]
Circ-WDR77	miR-124/FGF2 signaling regulation	miR-124; FGF2	[170]
Circ-ABCA1	miR-885-5p/ROCK2 axis control in VSMCs	miR-885-5p; ROCK2	[153]
Circ-LARP1B	cAMP pathway suppression via PDE4C induction	PDE4C; cAMP	[149]
Circ-SMAD3	hnRNPA1 degradation and p53γ splicing regulation	hnRNPA1; p53γ	[146]
Circ-TLK1	miR-513a-3p/KLF4 axis modulation	miR-513a-3p; KLF4	[154]
Circ-ZBTB46	hnRNPA2B1 stabilization and PTEN/AKT/mTOR pathway regulation	hnRNPA2B1; PTEN/AKT/mTOR	[134]

Abbreviations: NRG-1: neuregulin 1; TET2: ten-eleven translocation methylcytosine dioxygenase 2; DRP1: dynamin-related protein 1; ROS: reactive oxygen species; PTBP1: polypyrimidine tract binding protein 1; IGF1: insulin-like growth factor 1; FOXO1: forkhead box O1; FGF2: fibroblast growth factor 2; ROCK2: Rho-associated protein kinase 2; PDE4C: phosphodiesterase 4C; KLF4: Kruppel-like factor 4; hnRNPA1/2B1: heterogeneous nuclear ribonucleoproteins A1/A2B1; PTEN: phosphatase and tensin homolog.

## Data Availability

No new data were created or analyzed in this study.
